# Human CD4^+^ T-Cells: A Role for Low-Affinity Fc Receptors

**DOI:** 10.3389/fimmu.2016.00215

**Published:** 2016-06-01

**Authors:** Anil K. Chauhan

**Affiliations:** ^1^Division of Adult and Pediatric Rheumatology, Saint Louis University School of Medicine, St. Louis, MO, USA

**Keywords:** Fc-receptors, T-lymphocytes, autoimmunity, toll-like receptors, epigenetics

## Abstract

Both lymphoid and myeloid cells express Fc receptors (FcRs). Low-affinity FcRs engage circulating immune complexes, which results in the cellular activation and pro-inflammatory cytokine production. FcRs participate in the internalization, transport, and/or recycling of antibodies and antigens. Cytosolic FcRs also route these proteins to proteasomes and antigen-presentation pathways. Non-activated CD4^+^ T-cells do not express FcRs. Once activated, naive CD4^+^ T-cells express FcγRIIIa, which, upon IC ligation, provide a costimulatory signal for the differentiation of these cells into effector cell population. FcγRIIIa present on CD4^+^ T-cell membrane could internalize nucleic acid-containing ICs and elicit a cross-talk with toll-like receptors. FcγRIIIa common γ-chain forms a heterodimer with the ζ-chain of T-cell receptor complex, suggesting a synergistic role for these receptors. This review first summarizes our current understanding of FcRs on CD4^+^ T-cells. Thereafter, I will attempt to correlate the findings from the recent literature on FcRs and propose a role for these receptors in modulating adaptive immune responses *via* TLR signaling, nucleic acid sensing, and epigenetic changes in CD4^+^ T-cells.

## Introduction

Immunoglobulin Fc receptors (FcRs) are expressed by many immune cells, and these receptors induce many diverse biological functions. Activating- and inhibitory-FcRs are expressed as pairs, and they govern the outcome of an immune response. Sandor and Lynch very early on showed that T-cell receptor (TCR)-activation results in the induced expression of FcαR, FcμR, and FcϵR in CD4^+^ T-cell clones (1). FcR common γ-chain (FcR-γ) is the ITAM-bearing signaling unit of FcϵRI, FcγRI, and FcγRIIIa (2). FcR-γ chain independently supports the complete development of peripheral CD4^+^ T-cells in mice lacking the TCR ζ-chain (3–5). The FcR-γ chain forms a heterodimer with the ζ-chain of TCR in CD4^+^ T-cells. Membrane-FcγRIIIa in these cells can signal using the ζ–ζ chain, γ–γ chain homodimers, or ζ–γ chain heterodimers. ζ-chain deletion is not a lethal event for CD4^+^ T-cell development, suggesting an alternative-signaling pathway using the FcR-γ chain. In CD4^+^ T-cells, the FcR-γ chain engages Syk kinase for signaling (6). Syk is a ZAP-70 homolog that successfully substitutes for ZAP-70 kinase activity (7). Upon phosphorylation by FcR-γ chain, Syk provides a distinct and a stronger signal than the ZAP-70-ζ-chain of TCR complex (7). NK T-cells express both the ζ-chain and FcR-γ chain. Both of these signaling proteins associate with FcγRIIIa in human NK T-cells; however, in mice, it is only the FcR-γ chain that can associate with FcγRIII (8). In NK T-cells, the increased expression of ζ-chain downregulates FcγRIIIa expression (9). These studies suggest a regulatory role for these signaling proteins.

**Figure 1:**
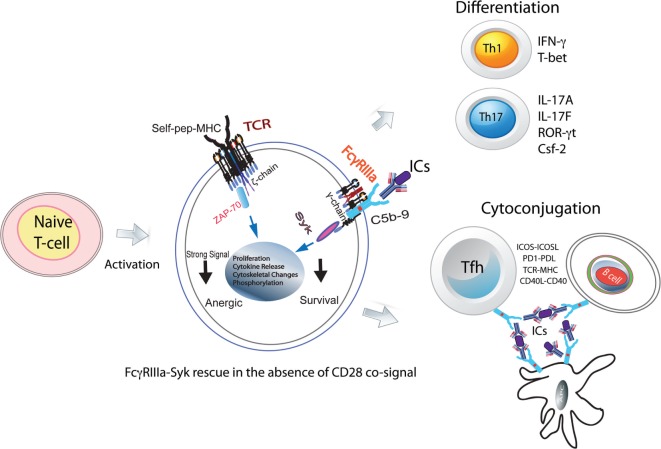
**FcγRIIIa–pSyk signal in CD4^+^ T-cell responses**. Activated naive CD4^+^ T-cells express FcγRIIIa and provide a costimulatory signal. In the absence of CD28 costimulation, FcγRIIIa ligation by ICs rescues naive CD4^+^ T-cells from becoming anergic. These cells show Syk phosphorylation, proliferation, cytoskeletal changes, and cytokine production. In the presence of instructive cytokine signals, these cells differentiate into Th1, Th2, and Tfh cells. In GCs, FcRs *via* ICs will hold together Tfh, B-cells, and FDCs forming stable cyto-conjugates.

Thus far, a role for low-affinity FcRs in CD4^+^ T-cell responses has not been envisioned, despite a number of early studies supporting the presence of these receptors on such cells. Of special interest is how positive costimulation from the FcγRIIIa–pSyk signal could alter CD4^+^ T-cell responses, which, thereby, contribute to tolerance breakdown (10, 11). Systemic lupus erythematosus (SLE) is a classical autoimmune manifestation and is a good model to address these questions since the disease pathology is driven by ICs, the primary ligand for FcγRIIIa (12). In addition, SLE is associated with a hyperactive T-cell response and the presence of autoantibodies that form ICs. Enhanced Th1 and Th17 CD4^+^ T-cell responses are a hallmark of SLE pathology. An indirect role for FcRs in the Th1 response has also been proposed (13). ICs are present on the membrane of subcapsular sinus macrophages and are not phagolysed. Intact ICs are transferred from the plasma membrane of antigen-presenting cells (APCs) to the B-cell surface (14–16). In germinal centers (GCs), this makes ICs available on APC and B-cell plasma membranes, which are accessible to participate directly in the CD4^+^ T-cell differentiation upon contact with naive CD4^+^ T-cells (Figure 1). Furthermore, ICs could also facilitate formation of cyto-conjugates of CD4^+^ T-cells with other cells expressing FcRs (Figure 1).

The ICs formed by nucleic acids (DNA/RNA) and autoantibodies are pathogenic and trigger TLR signaling and nucleic acid sensing (17). In SLE, FcγRIIa internalizes DNA/RNA-ICs in plasmacytoid dendritic cells (pDCs), which result in a type 1 IFN response, a key driver of SLE disease pathology (18). Even though CD4^+^ T-cells demonstrate TLR signaling in an autoimmune response, the mechanisms for the delivery of nucleic acids to cytosol are unknown (19, 20). Nucleic acid sensors in innate cells drive IFN responses that contribute to autoimmune pathology. Data are emerging that link both TLR proteins and DNA sensors in the development of CD4^+^ T-cell effector responses (21–23).

This review will summarize the literature supporting the presence of FcRs on CD4^+^ T-cells, and further makes a case for FcγRIIIa–pSyk signaling in the modulation of TLR responses and epigenetic changes in the human peripheral CD4^+^ T-cells.

## FcRs on CD4^+^ T-Cells

The expression of FcRs and their role in CD4^+^ T-cell-mediated adaptive immune responses is controversial. Several groups have argued for the lack of low-affinity FcRs on CD4^+^ T-cells (2, 24). This is likely true for non-activated CD4^+^ T-cells, and these cells do not contribute to the disease pathology. However, once activated, CD4^+^ T-cells express robust amounts of FcγRIIIa receptors, which is an activation-induced response (10). The activation signal that triggers the expression of FγRIIIa in activated cells remains unknown. FcγRIIIa was initially reportedly observed in a small number of peripheral T-cells in healthy individuals (1, 25). Upon binding to FcRs on T-cells, IgM triggers a helper function, while ICs binding provides a suppressor function (26). Two previous studies have also shown an immunoregulatory role for FcR-bearing T-cells in a B-cell-mediated immune response (27, 28). A close relationship between FcR expression and cellular activation *via* the CD3–TCR complex was also documented (28). A stringent and narrow window during which FcRs are expressed on CD4^+^ T-cells suggest a possible regulatory role for FcRs in adaptive immune responses, and FcR signaling may serve as a checkpoint for the development of T effector cells (29). FcR and TCR comigrate on the T-cell membrane, suggesting a synergism in signaling pathways (1, 30, 31). FcR preferentially colocalizes with TCR into the zone of contact formed between B- and T-cells during cognate-driven cyto-conjugation (1). In trogocytosis, CD4^+^ T-cells capture both external membrane FcγRIIIa and FcR-γ chain from the APC expressing FcγR. However, this receptor transfer/capture of FcRs by T-cells is not capable of triggering a functional response (32). FcγRIIIa-mediated signaling in NK T-cells differs from CD4^+^ T-cells for the production of cytokines, which further suggest a divergent role for FcR in CD4^+^ T-cells (33). Sandor and Lynch proposed an “avoidance hypothesis,” where a signal in T-cells *via* FcγRIII might occur in the presence of antigens and specific antibodies (1). Naive CD4^+^ T-cells activated *via* ICs ligation of FcγRIIIa show a limited clonal expansion, suggesting a potential contribution from antigenic peptides in the ICs. ZAP-70-deficient patients express high levels of Syk, which is activated from FcR-γ chain phosphorylation, and it plays a distinct role in transducing TCR-mediated signal (34). These findings suggest a role for FcγRIIIa signaling *via* Syk (Figure 1). Syk is a key player in CD4^+^ T-cell activation in SLE and is currently a therapeutic target (35, 36).

## FcRs and T-Cell Responses

In order for naive CD4^+^ T-cells to differentiate into effector cells, it requires two signals: (1) engagement of TCR by peptide–MHC and (2) a cosignal from CD28 upon the ligation by CD80/CD86 expressed by APCs (37). A third signal from cytokines determines whether these cells differentiate into effector Th1, Th2, Th17, or regulatory T-cells (Treg) cells. These populations maintain and regulate immune homeostasis. Both Th1 and Th17 cells cause and sustain tissue damage, while Tregs suppress these pro-inflammatory cells. Some of the early studies have implicated FcRs in the development of suppressor T-cells, now recognized as Tregs (26). Thus, it is important to recognize the role of FcγRIIIa signal as an additional positive costimulatory signal for CD4^+^ T-cell differentiation.

The secondary adaptive immune responses are fast and robust due to rapid expansion of antigen-specific lymphocytes. FcRs facilitate these responses by binding to ICs formed by affinity-matured autoantibodies against autoantigens. In autoimmunity, aberrant CD4^+^ T-cell responses are frequently observed, which are accompanied by autoantibody production and the IC formation. CD3 ligation in the absence of CD28 signal makes naive CD4^+^ T-cells anergic. However, in an autoimmune background, naive CD4^+^ T-cells bypass the CD28 signal requirement for activation (Figure 1). The underlying mechanism for this activation in the absence of CD28 signal is unknown (38). Unlike mice, where naive CD4^+^ T-cells are produced in the thymus, in humans, 90% of these cells are produced in the periphery from proliferation (39). Thus, a likely scenario is that, in humans, peripheral CD4^+^CD45RA^+^ (naive) T-cells have encountered antigen in the periphery and hence are different than those observed in mouse (39). In multiple sclerosis, differences in naive CD4^+^ T-cell biology, notably of TCR and TLR signaling, have identified patients prone to more rapid conversion to secondary progression (40). Nano-LC/MS/MS analysis of ICs obtained from SLE patients show the presence of 40–250 antigenic peptides. What role these IC peptides play in the T-cell activation is not clear (41). Human naive CD4^+^ T-cells activated *in vitro* by the suboptimal engagement of CD3 and costimulated either *via* CD28 cosignaling or with ICs in the presence of non-lytic C5b-9 induce FcγRIIIa expression (10). Upon FcγRIIIa engagement by SLE-ICs, these cells produce high amounts of IFN-γ and IL-17A (11). The FcγRIII-mediated production of IFN-γ is also observed in NK T-cells (9). Several mechanisms contribute to the peripheral tolerance breakdown, which results in an autoimmune response (42). TCR signal strength is one contributor to tolerance breakdown. FcγRIIIa can provide an additional positive signal to the TCR for overcoming the threshold for tolerance breakdown. Thus, specific inhibition of FcγRIIIa signal in CD4^+^ T-cells provides an attractive therapeutic target. Costimulatory pathways influence the outcome of T-cell stimulation and are central to the maintenance of immune tolerance (42). In the early phase of antigenic challenge from pathogens or self-antigens, activating cosignals CD28 and ICOS drive an immune expansion. Once the threat from the invading pathogens is over, the immune contraction phase is initiated by the appearance and expansion of the negative costimulatory proteins PD1 and CTLA4. In an autoimmune response, the FcγRIIIa-mediated signal, which is an additional positive ITAM signal, can drive immune expansion. This will lead to perpetual expansion of the immune response such as that observed in autoimmunity. Syk phosphorylation is observed in those SLE CD4^+^ T-cells that also produce IFN-γ and IL-17A cytokines (11). FcγRIIIa cosignaling drives the differentiation of naive CD4^+^ T-cells into Th1, Th17, and Tfh effector populations (10, 11, 43). The Th17 cells produced by an FcγRIIIa signal show markers of terminal differentiation that are associated with a pathogenic Th17 population (11). *In vitro*, FcγRIIIa ligation by ICs on naive CD4^+^ T-cells induces ICOS expression both in human and mouse cells. ICOS^+^CD4^+^ T-cells in SLE patients bind to labeled ICs, suggesting FcγRIIIa coexpression (11). However, cells expressing high levels of PD1^high^ do not show pSyk, suggesting a role for PD1 in immune contraction *via* SHIP2 by dephosphorylating pSyk (11). IC formation is observed in several other disease pathologies, including cancer and infections. Past and recent literature suggests that FcγRIIIa is a crucial player for CD4^+^ T-cell responses during autoimmunity. In future, recognizing the precise mechanism of how FcγRIIIa-mediated signaling in CD4^+^ T-cells alters the adaptive immune responses will be critical for developing therapies that target CD4^+^ T-cell membrane proteins such as CTLA4, PD1, TLRs, and nucleic acid sensors.

## Cross-Talk Between TLRs and FcRs

**Figure 2:**
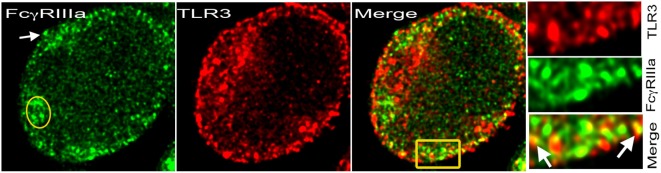
**TLR3 on cell membrane with FcγRIIIa**. Stimulated emission depletion (STED) confocal image of a human CD4^+^ T-cell activated with plate-bound anti-CD3 + ICs + sublytic C5b-9 post 48 h. FcγRIIIa recognized by binding of Alexa Fluor 488-labeled ICs (green) and TLR3 with monoclonal antibody-Alexa Fluor 594 (red). At the plasma membrane, both proteins colocalized in many spots. IC binding showed cytosolic FcγRIIIa (yellow oval) and on the plasma membrane (white arrow). Area amplified from merge shown in yellow rectangular.

TLR-dependent T-cell activation is observed in autoimmunity (19). The presence of FcRs on activated human CD4^+^ T-cells raises the possibility of their coengagement with either TCR or TLRs for signaling. Upon ligand engagement, TLRs trigger homo-or hetero-dimerization and recruit adaptor proteins to activate downstream signaling and transcriptional activation (44). Distinct signaling by synergistic engagement and cross-talk between FcRs and TLRs in immature DCs promote a Th17 response (45, 46). Such cross-talk between FcRs and TLRs expressed by CD4^+^ T-cells will result in an efficient inflammatory immune response and effector T-cell development (47). In B-cells, TLRs synergistically engage FcRs, which generate a distinct signal (47). Similar signaling events could occur in CD4^+^ T-cells that express FcRs. TLRs bind to pathogen-derived nucleic acids, which are taken up by the cells *via* endocytosis or autophagy and transferred to the endolysosomal compartment (48). Intracellular pathogenic challenge triggers the generation of Th1 and CD8^+^ T-cell responses, which develop from the engagement of TLRs by pathogen-associated molecular patterns (PAMPs) and produce IL-12, a cytokine that drives IFN-γ production in Th1 cells. Both ITAM and MyD88 signaling pathways converge after coactivation of FcRs and TLRs, resulting in an appropriate inflammatory response. Coactivation of FcRs by ICs and TLRs *via* damage-associated molecular pattern (DAMP) on infiltrating myeloid cells in joints of rheumatoid arthritis patients contributes to severity of the disease (47). ICs and PAMPs or DAMPs induce cross-talk and contribute to both the onset and the exacerbation of autoimmune disease (47). The nucleic acid-recognizing TLRs (NA-TLRs), also referred to as endosomal TLRs (TLR3, TLR7, TLR8, and TLR9), participate in an autoimmune response (20). The subcellular partitioning of TLRs, cytosolic vs. membrane, discriminates between self and altered non-self DNA and is a key mechanism for the development of autoimmunity (49). FcγRIIIa-mediated signal in CD4^+^ T-cells upregulates NA-TLRs, which then colocalize with FcγRIIIa, and some of these NA-TLRs move to the plasma membrane (Figure 2) (11). This is the evidence for FcR’s role in modulating TLR signaling in CD4^+^ T-cells. FcγRIIIa-mediated signaling in CD4^+^ T-cells also upregulates MyD88 and HMGB1, the two proteins that are critical for TLR signaling and nucleic acid sensing (11). DNA-containing ICs in B-cells and pDCs trigger HMGB1-mediated TLR9 activation that contributes to autoimmune pathology (50). TLR agonists have been used to study TLR signaling events in CD4^+^ T-cells (19). Surprisingly, various CpG oligodeoxynucleotides, a TLR9 ligand used to study both mouse and human CD4^+^ T-cells, show a costimulatory activity that promotes polarization of different Th subsets (51). TLR9-deficient lpr/lpr mice show a selective defect in autoantibody production (52). DAMPs induce inflammatory T cell responses either directly or indirectly by inducing cytokine production from innate cells. TLR signaling overcomes a rate-limiting chromatin barrier from histone-containing nucleosomes that bind DNA, suggesting its role in epigenetic modifications (53, 54). IFN-γ cytokine is a key player in TLR signaling and chromatin remodeling, and it is produced from FcγRIIIa signaling in CD4^+^ T-cells (10, 53). Observations over the last decade have also shown a DC subset-specific expression of PRRs and cytokines produced by these cells promote the differentiation of T-cells into effector populations (55). Emerging evidence suggests that both FcR and TLR signaling trigger CD4^+^ T-cell-mediated pro-inflammatory responses. How these signals together influence the development of effector T-cells and/or their contribution to the expansion of memory T-cell pool will be of interest.

## FcRs and Nucleic Acid Sensing

Nucleic acid-recognizing TLRs primarily reside intracellularly in endosomes. This prevents recognition of self nucleic acids by the host. Internalization of RNA- or DNA-containing ICs by FcγRIIa and subsequent delivery of nucleic acids to endosomes results in an inappropriate IFN response (20, 23). Nucleic acid reaches the cytosolic compartment either *via* viral infection or by uptake of DNA/RNA-ICs. Thus, FcRs are crucial for the delivery of nucleic acids to the cytosol, where DNA sensing occurs (22). Nucleic acid-sensing pathways are the therapeutic targets in both autoimmunity and cancers (56). High mobility group box 1 (HMGB1) protein is crucial for the efficient delivery of self nucleic acids to TLR-containing endosomes (50, 57). HMGB1 associates with DNA-ICs and stimulates cytokine production *via* the TLR9–MyD88 pathway in pDCs (50). HMGB1 uses TLR2 and TLR4 as cellular receptors and engages S100 calcium binding protein A12 (RAGE) to produce type 1 IFN (58). The mechanism and route for RNA/DNA delivery to endolysosome or to the ER in CD4^+^ T-cells are not yet recognized. Membrane FcγRIIIa on activated CD4^+^ T-cells provides a possible route and mode of RNA/DNA-ICs internalization and delivery of the nucleic acids to the endosomes (Figure 2).

Nucleic acids activate immune cells to induce inflammation and immunity (22). Both TLRs and retinoic acid-inducible gene 1 (RIG-1)-like helicases participate in nucleic acid recognition. Immune sensing of DNA is observed both in early innate as well as late adaptive immune responses (59, 60). Aberrant DNA, altered self-DNA, and cyclic dinucleotide sensing by signaling proteins in the cytosol trigger DNA-signaling pathways. DNA-ICs and nucleosomes are closely associated with the development of inflammation in SLE. DNA-sensing proteins co-operate with TLRs to mount the immune challenge and discriminate against damaged DNA. ICs that stimulate B-cells contain DNA that is hypomethylated and such ICs modulate T-cell responses (61). Members of the DExD/H box helicase family of proteins, such as RIG-1, interferon inducible helicase C (IFIH1/MDA5), and RIG-1-like receptor (LGP2) recognize cytosolic ssRNA and dsRNA and signal through mitochondrial antiviral signaling proteins to activate interferon regulatory factor (IRF) 1, IRF3, IRF7, and NF-κB. These, in turn, trigger expression of IFN-α and other pro-inflammatory cytokines (21). FcγRIIIa engagement by ICs in CD4^+^ T-cells enhances the expression of both IRF3 and IRF7. FcγRIIIa expression by CD4^+^ T-cells opens up the possibility that the DNA-ICs bind to PHYIN (pyrin and HIN200) domain-containing family protein absent in melanoma 2 (AIM2) and to apoptosis-related speck protein (ASC), leading to the formation of inflammasome-like structures producing IL-1β in CD4^+^ T-cells. IC stimulation of human naive CD4^+^ T-cells *via* FcγRIIIa induces production of IL-1β (11). Thus, the expression of FcγRIIIa on activated CD4^+^ T-cells could be a crucial participant in the nucleic acid-signaling pathway.

## Epigenetic Changes in CD4^+^ T-Cells from FcγRIIIa–Syk Signaling

Epigenetic modifications are crucial for the differentiation and maintenance of CD4^+^ helper T-cell subsets. Acetylation and methylation of histones, which are considered epigenetic marks, are observed in SLE pathology. Both *ifng* and *il17a* promoters show epigenetic marks. Hypomethylation of *il4* and *il6* promoters are associated with disease activity (62). Even though a role for epigenetics in the differentiation of CD4^+^ T-cell helper subsets is recognized, what drives these changes is unknown. In particular, it will be of interest to examine the mechanisms that drive epigenetic changes in the Bcl6 locus. Bcl6 is a transcription factor associated with the Tfh lineage that exhibits H3K4me3 marks, suggesting gene upregulation. Those genes that cause modification to histones and DNA show marked upregulation by FγRIIIa–pSyk signaling in naive CD4^+^ T-cells. This signal also drives the differentiation of Th1, Th17, and Tfh subsets (11, 43, 63). Our analysis of five donors for the genes associated with epigenetic chromatin modification showed a correlation among DNA (cytosine-5-)-methyltransferase 1 (DNMT1), retinoblastoma-binding protein 7 (RBBP7), chromodomain helicase DNA-binding protein 4 (CHD4), and methyl-CpG-binding domain protein 2 (MBD2) (63). These enzymes are part of a histone deacetylase complex and are upregulated by the FcγRIIIa–pSyk signal. The SET domain-containing lysine methyltransferases (SETD)-7 and SETD-2, which generate an H3K4me1, and SETD-1 that generates a H3K9 methyl mark, were also significantly upregulated by FcγRIIIa–pSyk cosignaling compared to the canonical CD28 cosignal (63). H3K4me1 is enriched at poised or active genes and shown to be a marker of active enhancers in combination with H3K27ac and p300 binding (64). H3K27 hypomethylation and H3K18 hypermethylation are observed in activated naive CD4^+^ T cells and in SLE T-cells (65). These findings suggest that by upregulating DNA modification enzymes, the FcγRIIIa–pSyk signal causes epigenetic changes in human CD4^+^ T-cells. In Th1 cells, the *ifng* locus shows permissive histone modifications and DNA demethylation (66). In the promoter regions of 14,495 genes in SLE, CD4^+^ T cells, 236 were hypomethylated and 105 were hypermethylated (67). A profound hypomethylation of genes regulated by type I IFN was observed in genome-wide DNA methylation analysis (68). Methylation changes in SLE persist beyond flares for several months (68). SLE patients demonstrate the elevated presence of complement-opsonized ICs that engage membrane FcγRIIIa. Thus, it is plausible that the FcγRIIIa–pSyk signal in CD4^+^ T-cells contributes to the modifications observed in the *ifng* and *il17a* promoters. DNA methyltransferase, Dnmt3a, establishes a genetically silent chromatin structure at the regulatory region of *ifng* locus by methylating DNA. It has been proposed that methylation at −53 CpG by Dnmt3a suppresses IFN-γ transcription during Th2 development (69). FcγRIIIa–pSyk signaling suppresses Dnmt3a expression compared to CD28 signaling in CD4^+^ T-cells. This −53 region is also the preferred binding site for activation transcription factor (ATF)2. In our study, the FcγRIIIa–Syk signal significantly upregulated the ATF2 gene expression, suggesting a possible role in increased IFN-γ production (11). KAT6A is another lysine-transferase that was significantly upregulated by FcγRIIIa–pSyk signaling. This gene is suggested to act as a coactivator of RUNX1, which drives Th17 differentiation. Children with a mutation in KAT6A show developmental disorders and cognitive defects (www.Chloekat6a.org). Decrease in DNMT expression results in hypomethylation of promoters of SLE-associated genes, which drives their overexpression. A positive correlation with DNMT1 and MBD2 expression is observed with disease activity in SLE patients (70, 71). Epigenetic changes not only regulate the differentiation of CD4^+^ T-cells but also TLR signaling (72). CD4^+^ T cells express TLR4 (not a DNA sensor), which drives epigenetic regulation of the TNF-α promoter (73). Also, the pan-histone deacetylase inhibitor LBH589 represses cytokines IL-6, IL-10, IL-12, and IL-23 (74). Treatment of CD4^+^ T-cells with demethylating agents (hydrazine, procainamide, and 5-AzaC) renders them autoreactive. Adoptive transfer of such cells in mice causes them to produce anti-dsDNA antibodies and develop IC glomerulonephritis (75). These studies document a role for epigenetics in autoimmune pathology and data from our laboratory showed modulation of several enzymes that cause epigenetic modifications by FcγRIIIa–pSyk signaling (63). It will be important to further investigate these mechanisms to understand and enhance the efficacy of demethylating agents for SLE therapy.

## Concluding Remarks

Even though earlier studies documented the presence of low-affinity FcRs on CD4^+^ T-cells, neglect in examining the contribution of these receptors in CD4^+^ T-cell responses over the past two decades has hampered progress in establishing the contribution of FcRs to adaptive immune responses. Emerging data reconfirm some of the earlier findings that activated CD4^+^ T-cells not only express FcRs, but signaling *via* these receptors modulates adaptive immune responses. Engagement of FcRs by the ligand contributes to the development of CD4^+^ effector T-cell responses. Low-affinity FcRs are critical for innate immune responses, and their presence on CD4^+^ T-cells, cells of adaptive immunity, suggests their critical role in adaptive immunity. Cross-linking by complexed-Ig triggers proliferation of FcγRIIIa-bearing CD4^+^ T-cells *via* receptor dimerization. The internalization of RNA/DNA-ICs by FcγRIIIa^+^CD4^+^ T-cells by engaging TLRs triggers signaling *via* DNA sensors. Whether these signaling events contribute to the development of IFN signature and plasma cell development will be important to understand the underlying mechanism of autoimmune pathology. It will be of further significant interest to explore whether induced expression of TLR proteins by FcγRIIIa signaling generate a cross-talk with TLRs and enhance nucleic acid sensing. How these signals influence the fate of effector T-cells and contribute to the central memory pool is an important question. On memory recall, such cells will be able to provide B-cell help and drive them to differentiate into plasma B-cells. Understanding of the interactions among FcRs, TLRs, and/or TCR will assist in explaining autoimmune pathology.

## Author Contributions

The author confirms being the sole contributor of this work and approved it for publication.

## Conflict of Interest Statement

The author declares that the research was conducted in the absence of any commercial or financial relationships that could be construed as a potential conflict of interest.
